# ALKBH5 Inhibits YTHDF2‐m6A‐Mediated Degradation of RCN1 mRNA to Promote Keloid Formation by Activating IRE1α‐XBP1‐Mediated ER Stress

**DOI:** 10.1111/jocd.70177

**Published:** 2025-04-11

**Authors:** Min Shi, Lu Zhang, Fangfang Bi, Zhuo Zhou

**Affiliations:** ^1^ School of Medicine, Xi'an Peihua University Xi'an Shaanxi China; ^2^ Department of Obstetrics and Gynecology Northwest University First Hospital Xi'an Shaanxi China

**Keywords:** ALKBH5, fibroblasts, IRE1α‐XBP1, keloid, RCN1

## Abstract

**Background:**

Reticulocalbin 1 (RCN1) was reported to be upregulated in keloid, but its molecular mechanism remains unclear. The aim of this study is to investigate the role of RCN1 in keloid.

**Methods:**

The expression of RCN1 was detected in keloid tissues. Keloid fibroblasts were transfected with RCN1 overexpression vector. Cell viability, collagen production, apoptosis, and cell invasion were measured. Then, the m6A modification level of RCN1 mRNA was detected by methylated RNA immunoprecipitation (MeRIP), and the effect of overexpression of ALKB homolog5 (ALKBH5) on the m6A modification level of RCN1 mRNA was evaluated. Subsequently, the relationship between RCN1 and XBP1 was verified by co‐immunoprecipitation (Co‐IP) assay. pcDNA‐RCN1 and XBP1 shRNA were transfected into keloid fibroblasts to for reversal experiments, and changes in the endoplasmic reticulum (ER) structure of keloid fibroblasts were observed by transmission electron microscopy (TEM). Finally, we established a mouse keloid model and injected mice with the RCN1 shRNA lentiviral vectors to monitor the keloid formation in mice.

**Results:**

RCN1 was highly expressed in keloid tissues and keloid fibroblasts. Overexpression of RCN1 significantly increased keloid fibroblast viability, collagen production, and invasion, but inhibited cell apoptosis. ALKBH5 upregulated RCN1 expression by reducing m6A‐YTHDF2‐mediated degradation of RCN1 mRNA, and RCN1 knockdown reversed the promoting effect of ALKBH5 overexpression on cell viability collagen production and invasion, and the inhibitory effect of ALKBH5 overexpression on apoptosis in keloid fibroblasts. Moreover, overexpression of RCN1 significantly upregulated the protein levels of XBP1, GRP78, and IRE1α, and promoted ER stress in keloid fibroblasts, but this change was eliminated by sh‐XBP1 intervention. In vivo experiments showed that knockdown of RCN1 significantly inhibited keloid formation by alleviating cell apoptosis and ER stress in mice.

**Conclusion:**

Our data revealed that RCN1 was upregulated by ALKBH5 to promote keloid formation by activating IRE1α‐XBP1‐mediated ER stress, RCN1 may be a potential biomarker for treatment of keloid.

## 1 Introduction

Keloid is one of the most common pathological scars produced from deregulated wound healing that occurs upon skin tissue damage [1, 2]. It is characterized by overproduction of extracellular matrix, excessive keloid fibroblasts proliferation, overexpression of growth factors and anomalous disposition of collagen fibers [3, 4]. The main manifestations of keloid are prominent in normal skin, with various shapes, reddish color, hard and benign masses, and patients suffer from long‐term symptoms, such as itching and pain, which seriously affect the physical and mental health of patients [5, 6]. Currently, keloid was considered to be a benign skin tumor, not fatal, but has a high recurrence rate [7]. Because there are still no fully curative treatments, it is of utmost importance to comprehend the pathogenesis of keloid and investigate precise and efficacious approaches for their treatment.

Recent research showed that the ER in keloid tissues appeared thickened and with a rough and irregular surface compared to that in normal skin tissues, indicating that ER stress was associated with keloid [8]. When misfolded proteins accumulate acceptable levels in the ER beyond, the loss of ER protein homeostasis triggers an intracellular signaling pathway called unfolded protein response (UPR). In mammalian cells, UPR is initiated by three single pass ER transmembrane proteins, inositol‐demanding enzyme 1 α (IRE1α), protein kinase R (PRK)—like ER kinase (PERK), and transcription activator 6 (ATF6) [9]. XBP1 serves as a transcription factor for IRE1 substrate, and IRE1α regulates endoplasmic reticulum stress by modulating XBP1 splicing [10]. Moreover, a previous study showed that XBP1 was upregulated in keloid and IRE1α/XBP1 pathway was activated in keloid fibroblasts. Knockdown of IRE1α inhibited the expression of TGF‐β1, and the cell viability and cell cycle progression of keloid tissue are also significantly affected [11]. Boyko showed that inhibition of IRE1α could reduce keloid formation in wound healing mice and was upregulated during the proliferative phase [12].

RCN1 is a member of the CREC family located in ER. It consists of an ER‐retention motif, HDEL, and six EF‐hand motifs in its carboxyl‐terminal sequence [13]. Bioinformatics analysis showed that RCN1 carried out a number of functional activities, including calcium homeostasis and secretory cargo sorting and was upregulated in keloid [14]. Moreover, RCN1 was upregulated in dermal fibroblasts from patients with systemic sclerosis, therefore RCN1 can serve as a key regulator in fibroblasts [15]. These results displayed that RCN1 is closely related to keloid fibroblasts, and may be involved in keloid progression as a regulator, but its specific regulatory mechanism is unclear.

N6‐methyladenosine (m6A), the most common and abundant RNA modification in eukaryotes, played a crucial role in many biological processes and attracts increasing attention [16]. The process of m6A modification is dynamically reversible and is regulated by several writers (methyltransferases), erasers (demethylases), and readers (effector proteins), which participate in mRNA translation, splicing, transport, stabilization, and degradation [17]. A recent study showed that m6A modification is associated with keloid and can serve as a potential target for keloid treatment [18]. For example, Fu et al. [19] showed that ZC3H13 accelerates keloid formation by mediating m6A modification of HIPK2. Ren et al. [20] suggested that KIAA1429 regulated TGF‐β1 m6A modification, maintained TGF‐β1 mRNA stability, and participated in regulating keloid formation. In addition, Lin et al. [21] indicated that keloid fibroblasts with high m6A modification activated the Wnt/β‐catenin signaling pathway, which might promote keloid formation. ALKBH5 is a well‐known m6A demethylase (also called m6A “eraser”) and plays a role in regulating different target genes or m6A modifications in different regions of the same gene [22]. It's proven that m6A demethylase FTO promotes the development of scar tissue by regulating COL1A1 m6A modification, maintaining mRNA stability and upregulating COL1A1 expression [23]. However, the function and mechanism of ALKBH5 in keloid remain unclear. YTHDF families function as the main m6A readers by binding to methylated RNA and mediating specific function [24]. It's reported that YTHDF2 played important roles in many cancers, such as gastric cancer and liver cancer, but few studies extensively explored in keloid [25, 26].

In this study, we elucidated the role of RCN1 in the progression of keloid and its specific molecular mechanism by constructing keloid models in vivo and in vitro, aiming to identify potential targets for the treatment of keloid.

## 2 Materials and Methods

### 2.1 Tissue Specimen

Twenty patients (13 males and 7 females) were pathologically confirmed as keloid specimens (without the epidermis), with an age range of 24–67 years, average age of 43 years, and illness duration between 1 and 8 years (Table 1). All subjects signed the informed consent forms. All experimental protocols were approved by the Ethics Committee of the Northwest University First Hospital (NUFHLAC‐2022‐056), and all participants signed informed consent. These methods were carried out in accordance with relevant guidelines and regulations.

**TABLE 1:** Twenty patients information including age, gender and pathological feature.

Patient	Age (years)	Gender	Site	Tissue type	Diagnosis	Duration (years)
1	64	F	Left shoulder	Keloid	Large lump, irregular, with obvious telangiectasia visible on the surface, dark color	6
2	58	F	Chest	Keloid	The central mass in the chest area repeatedly breaks and scabs, causing itching, pain, and discomfort	1
3	33	M	Left ear lobe	Keloid	The protrusion behind the earlobe is about 0.5 cm; Uniform red color, hard texture, no rupture	2
4	38	M	colpus	Keloid	Redness and local itching	1
5	26	F	Left temporal region	Keloid	Epidermal atrophy and thinning, dense and irregularly arranged collagen fiber bundles in the dermis	1
6	67	F	Left shoulder	Keloid	Skin redness, high oxygen levels, accompanied by pain	3
7	62	M	right breast	Keloid	Large area, multiple, and wide keloid base, varying thickness	1
8	48	F	Chest and left shoulder	Keloid	Keloid nodules appeared on the surgical incision of the chest and left shoulder, with hard texture and accompanied by itching	3
9	30	M	Right ear	Keloid	Itchy, red, high and painful skin	2
10	24	F	Double jaw	Keloid	Visible nodules of varying sizes, raised above the surface of the skin, with a hard texture and a bright red color	8
11	25	F	Right ear	Keloid	Uniform red color, hard texture, no rupture	2
12	33	F	Left shoulder	Keloid	Multiple, densely distributed, obviously raised on the skin surface, round or irregular, varying in size, purplish red	3
13	32	F	Left shoulder	Keloid	Keloid are hard, tough, and congested nodular, linear	4
14	26	M	Double ear lobe	Keloid	Keloid in the ear lobe was caused by ear piercing, and the texture of the earlobe scar was hard, and the blood vessels were thin and not abundant	1
15	28	M	Left breast	Keloid	Red or dark red in color, in the form of stripes, butterflies, circles, irregular shapes	2
16	62	F	Left shoulder	Keloid	Large lump, irregular, with obvious telangiectasia visible on the surface, dark color	3
17	60	F	Right breast	Keloid	Keloid on the chest are about 20 × 20 cm, protruding from the body surface	3
18	58	F	Right shoulder	Keloid	Red or dark red in color, accompanied by itching or stabbing pain, with some clearly extending capillaries outward	1
19	53	F	Double jaw	Keloid	Visible nodules of varying sizes, raised above the surface of the skin, with a hard texture and a bright red color	1
20	33	M	Left breast	Keloid	Varying sizes, all of which are higher than the body surface	2

### 2.2 Fibroblast Isolation and Culture

Keloid fibroblasts and human normal skin fibroblasts were isolated from keloid skin and normal skin samples, as described previously [9, 19]. Keloid tissues were washed with PBS, and epidermis and subcutaneous adipose tissue were removed. Subsequently, specimens were cut into 1 to 3 mm^3^ pieces. After digestion, the suspension was filtered and centrifuged at 1500 rpm for 5 min. Keloid fibroblasts were cultured in Dulbecco's Modified Eagle's medium supplemented with 10% fetal bovine serum (Gibco, Grand Island, NY, USA), penicillin, and streptomycin in an atmosphere containing 5% CO_2_ at 37°C.

### 2.3 Transfections

Overexpression plasmids of RCN1 (pcDNA‐RCN1), ALKBH5 (pcDNA‐ALKBH5), YTHDF2 (pcDNA‐YTHDF2), short hairpin RNA (RCN1 shRNA), XBP1(XBP1 shRNA), and the corresponding negative control (NC‐shRNA and vector) were purchased from Sangon (Shanghai, China). Lipofectamine 3000 transfection reagent (Invitrogen, USA) was used for cell transfection according to the manufacturer's instruction.

### 2.4 RNA Extraction and qRT‐PCR

TRIzol (Solarbio, Beijing, China) was used to extract total RNA from cultured normal fibroblasts and keloid fibroblasts. RNA concentrations were measured using the Nano Drop 2000 instrument (Bio‐Rad, Hercules, CA, USA). The complementary DNAs (cDNAs) was subsequently synthesized from 1 μg of total RNA using an RNA Reverse Transcription Kit (APExBIO, Houston, TX, USA). Then, a quantitative reverse transcription polymerase chain reaction (qRT‐PCR) experiment was performed using a SYBR Green PCR Kit (Cat: 4368711, TaKaRa, Tokyo, Japan). The reaction conditions as follows: denaturation at 95°C for 3 min, followed by 35 cycles at 94°C for 15 s, 55°C for 25 s, 72°C for 30 s. Tubulin was used as internal control. The mRNA expression levels were determined using the standard 2^−ΔΔ*Ct*^ method based on at least three biological replicates. The primer sequences of different genes are shown in Table 2.

**TABLE 2:** Primer sequences for real‐time PCR.

Gene	Species	Forward (5′‐3′)	Reverse (5′‐3′)
RCN1	Human	AAACGGGTGCAGAAAAGATACA	AGGTAGTAACCATAGGTGGCTT
ALKBH5	Human	CGCCGTCATCAACGACTACC	CGACACCCGAATAGGCTTG
α‐SMA	Human	ACTGCCTTGGTGTGTGACAA	CACCATCACCCCCTGATGTC
COL1A1	Human	CACCATCACCCCCTGATGTCAAGA	AGCAGACTGGCAACCTCAAGA
YTHDF2	Human	CAGGCAAGGCCGAATAATGC	TCTCCGTTGCTCAGTTGTCC
XBP1	Human	GTAAGAAATATTACTATAA	AGTAAGAAATATTACTATA
Tubulin	Human	GAACCTGAACCGCCTGATTG	GACCAGAGGGAAGTGGATACG

### 2.5 Western Blotting

RIPA buffer (Beyotime, Shanghai, China) was used to extract total protein, and BCA protein assay kit (Beyotime, Shanghai, China) was performed to quantify protein concentrations. The protein was separated on a 10% sodium dodecyl sulfate‐polyacrylamide gel electrophoresis (SDS‐PAGE) gel, and then transferred to polyvinylidene fluoride (PVDF) membranes (Membrane Solutions, Nantong, China). After blocking with 5% non‐fat milk for 2 h, the transferred membranes were incubated with primary antibodies at 4°C overnight. The primary antibodies used in this study were as follows: ALKBH5 (1:1000, ab195377, Abcam), RCN1 (1:1000, ab210404, Abcam), IRE1α (1:1000; ab48187, Abcam), XBP1 (1:500; ab37152, Abcam), GRP78 (1:1000, ab21685, Abcam), YTHDF2 (1:2000, ab220163, Abcam), α‐SMA (1:1000, ab240678, Abcam), COL1A1 (1:500, ab138492, Abcam) and Tubulin (1:1000, ab18207, Abcam). Then the membranes were incubated with horseradish peroxidase (HRP)‐conjugated goat anti‐mouse secondary antibody for 1 h at 4°C. The bands were visualized with an ECL kit (Thermo, Waltham, MA, USA), and the gray level of bands was analyzed using Image J software.

### 2.6 MTT Assay

The proliferation of keloid fibroblasts was assessed by MTT assay. After transfection for 24 h, keloid fibroblasts were harvested and seeded in 96‐well plates. Another blank control group was set up, and only cell culture medium was added. After 12, 24, 48, and 72 h of culture, 5 mg/mL MTT (10 μL, Zeye Biotechnology) was added to each well, and the cells were incubated at 37°C for 4 h. After removing the MTT solution to dissolve the precipitates, dimethyl sulfoxide (DMSO) was added, and it was put in a constant temperature oscillation box and oscillated for 10 min at 37°C. The optical density (OD) of each well was assessed at a wavelength of the 490 nm using an automatic enzyme labeling instrument [27].

### 2.7 Cell Apoptosis

The apoptotic rate of fibroblasts was analyzed using an Annexin V‐FITC/PI apoptosis detection kit (BD Biosciences). Fibroblasts were seeded in 6‐well plates at a density of 1 × 10^6^ cells/well and incubated for 12 h at 37°C. The cells were then stained with 5 μL Annexin V‐FITC and 5 μL propidium iodide (PI) for 15 min in the dark at room temperature. Cell apoptosis was detected with Flow Cytometer (BD Biosciences, San 184 Jose, CA, USA) according to the manufacturer's instructions.

### 2.8 Methylated RNA Immunoprecipitation (MeRIP)‐qPCR

Total RNA from fibroblasts was isolated using the PolyATtract mRNA isolation systems (Promega, Madison, WI, USA). Then, mRNA was denatured at 70°C for 10 min, and 5 μL of anti‐m6A (1:1000, ab208577, Abcam) or anti‐IgG (1:500, ab172730, Abcam) were conjugated to protein A/G magnetic beads in IP buffer (150 mM NaCl, 10 mM Tris–HCL, and 0.1% NP‐40) for 2 h at 4°C. A total of 100 ng of RNA was incubated with antibodies in buffer containing RNase and protease inhibitors. m6A RNA was eluted with 6.7 mM N6‐methyladenosine 5′‐monophosphate sodium salt (Sigma, St. Louis, MO, USA) for 1 h at 4°C. Total RNA was eluted with elution buffer and purified by phenol chloroform extraction. The enrichment of m6A was determined by RT‐qPCR analysis. Fold enrichment was calculated as the 2^−ΔΔ*Ct*^ of the eluate relative to the input sample.

### 2.9 Co‐Immunoprecipitation (Co‐IP) Assay

After transfection, cells were washed twice with ice‐cold PBS and lysed by RIPA lysis buffer. The supernatant collected by centrifugation and incubated with RCN1 or XBP1 antibodies at 4°C overnight. Then added 100 μL of protein A agarose beads to capture the antigen–antibody complex, and slowly shook the mixture at 4°C overnight. The composite material was centrifuged at 4°C and 3000 rpm for 3 min. Next, the supernatant was discarded and the agarose beads were washed three times with ice‐cold PBS. Finally, the complex was boiled with protein loading buffer to free the antigens, antibodies and beads. After centrifugation, the supernatant was taken for electrophoresis to detect the expression of the interacting proteins.

### 2.10 Transmission Electron Microscopy

The samples were fixed in a fixative containing 2.5% glutaraldehyde and 3 mM CaCl_3_ in 0.1 M cacodylate buffer for 1 h at room temperature. Samples were washed briefly in distilled water, followed by gradual dehydration in a graded ethanol series of 70%, 90%, and 95% (15 min per stage), and then 100% (three times for 30 min each time). The dehydrated samples were then infiltrated with 100% propylene oxide, and embedded in an epoxy resin for 24 h. Ultrathin sections (50 nm) were prepared and mounted on copper grids. The grids were post‐stained with uranyl acetate and lead citrate. TEM was performed on a JEM‐1400 (JEOL, Tokyo, Japan) at 120 keV equipped with a CCD camera system (Ultra Scan, Los Angeles, CA, USA).

### 2.11 Establishment and Treatment of Keloid Model

Forty‐eight male/female BALB/c nude mice (aged 6–8 weeks) were obtained from Experimental Animal Center of the Northwest University First Hospital. The mice were housed under a light/dark (12:12) conditions with a humidity of 50% and a temperature of 25°C. Keloid specimens were freshly removed from the epidermis with sterile scissors and cut into uniform pieces (8 × 5 × 5 mm), with the rest collected as initial controls. Keloid pieces were then implanted into nude mice subcutaneously. The mice were divided into four groups (*n* = 12 per group): the sham group, KLD group, KLD + shRNA group, and the KLD + shRCN1 group. In addition, the mice of KLD + shRNA group and KLD + shRCN1 group were injected with empty vector or RCN1 lentiviral interference vector. Sham treated mice received an identical volume of physiological saline solution instead of RCN1‐shRNA. After 21 days, the mice were sacrificed by a lethal injection of sodium pentobarbital followed by exsanguination, tissue and blood were obtained simultaneously. All animal experiments were approved by the Ethics Committee of the Northwest University First Hospital (Xi'an, China, Ethical approval number: NUFHLAC‐2022‐056), and the experimental procedures were performed in accordance with the National Institutes of Health Guide for the Care and Use of Laboratory Animals.

### 2.12 Hematoxylin and Eosin (H&E) Staining

Paraffin‐embedded skin tissues were cut into sections and placed on slides. The tissue was cut into 4 μm sections and stained with hematoxylin (#BL735A‐1, Biosharp, Beijing, China) for 5 min, rinsed with water for 2 min, and then treated with ethanol for 10 s. The slides were stained with eosin (#BL735A‐2, Biosharp) and incubated for 10–15 s. The slides were degreased with xylene and dehydrated with different dilutions of ethanol (100%, 95%, 85%, 70%, 50%). An Olympus BX63 microscope (Olympus, Tokyo Japan) was used to observe five fields and acquire images of stained slides.

### 2.13 Statistical Analysis

Statistical analysis was performed with SPSS version 17.0 (SPSS Inc., Chicago, IL), and all quantitative data are presented as means ± SEM. Differences between two groups were compared using Student's *t*‐test when they had a normal distribution. One‐way analysis of variance (ANOVA) or Two‐way analysis of variance (ANOVA) followed by Tukey's HSD test was applied to evaluate the significance among multiple groups according to the data normal distribution and homogeneity of variances. The significance level was set as *p* < 0.05.

## 3 Results

### 3.1 RCN1 Was Highly Expressed in Keloid Tissues and Keloid Fibroblasts

**FIGURE 1:**
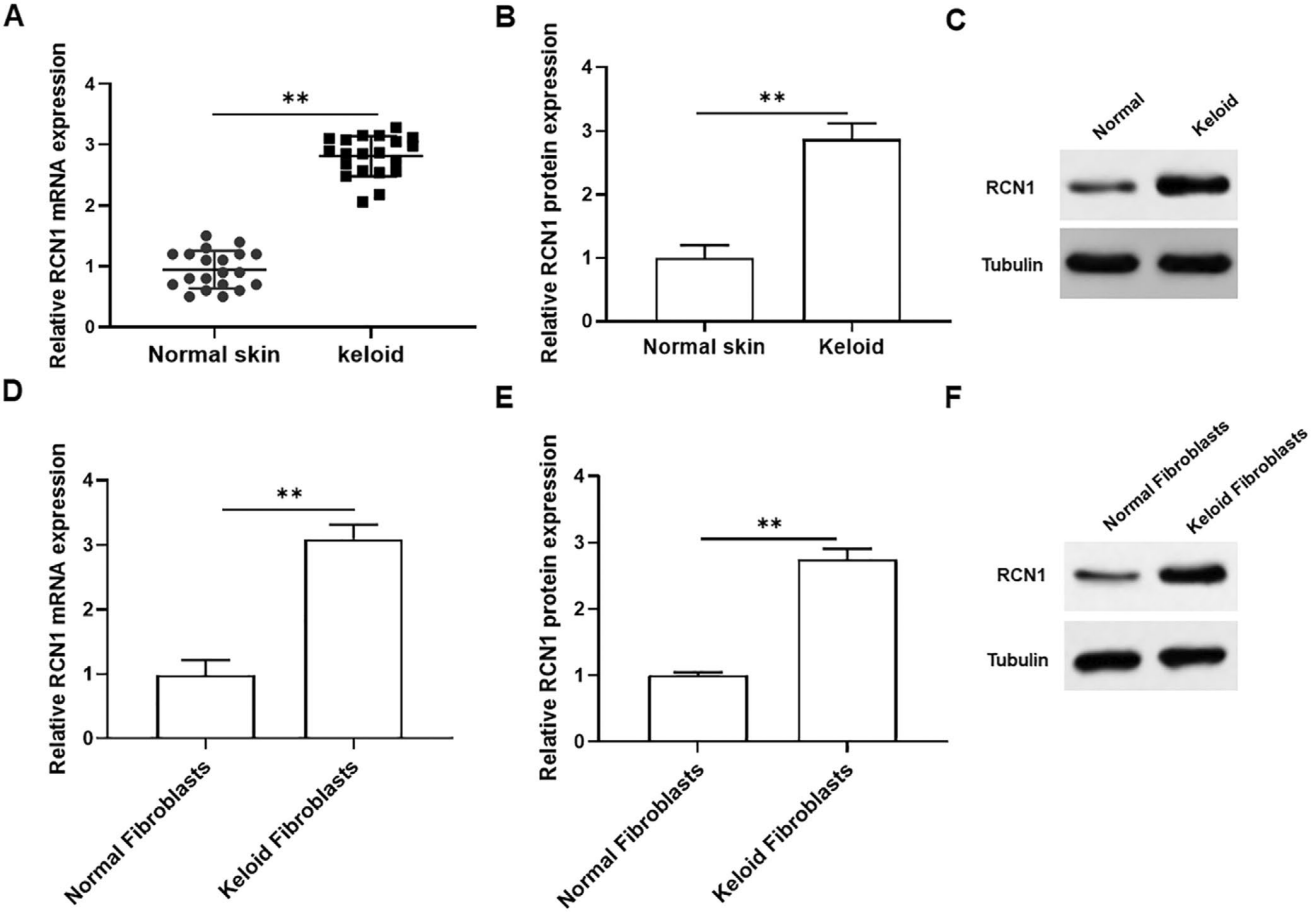
Expression of RCN1 in keloid tissues and keloid fibroblasts. (A) qRT‐PCR was used to detect RCN1 mRNA expression in keloid tissues and corresponding normal tissues. (B, C) RCN1 protein expression was detected by Western blotting in keloid tissues and corresponding normal tissues. (D) RCN1 mRNA expression was determined by qRT‐PCR in keloid fibroblasts and Normal fibroblasts. (E, F) Western blotting was used to detected RCN1 protein expression. All data are shown as means ± SEM. *N* = 5. Student's *t*‐test was used to calculate the differences between two groups **p < 0.01.

To investigate the role of RCN1 in keloid tissues and keloid fibroblasts, we first detected RCN1 expression in normal skin and keloid tissues. As presented in Figure 1A–C, the mRNA and protein levels of RCN1 were significantly elevated in keloid tissues compared to those in normal skin tissues. In addition, the mRNA and protein expression of RCN1 was significantly upregulated in keloid fibroblasts compared to that in normal fibroblasts (Figure 1D–F). Thus, we inferred that RCN1 might play a crucial role in keloid formation.

### 3.2 Overexpression of RCN1 Promoted the Viability, Invasion and Collagen Production of Keloid Fibroblasts, and Inhibited Cell Apoptosis

**FIGURE 2:**
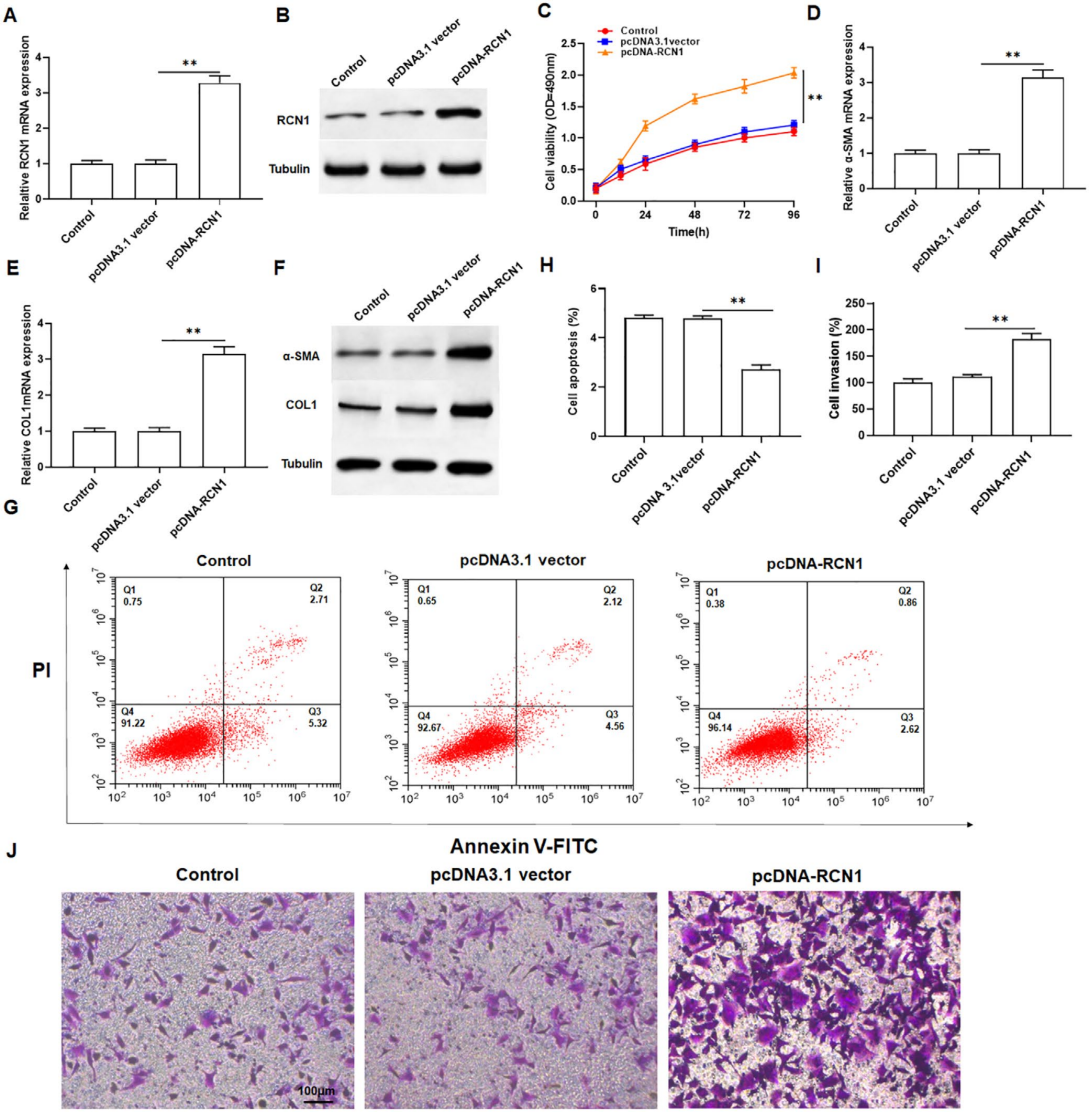
Effect of RCN1 overexpression on the behavior of keloid fibroblasts. Keloid fibroblasts were transfected with pcDNA‐RCN1 or pcDNA3.1 vector. (A, B) The mRNA and protein expression of RCN1 were detected by qRT‐PCR and Western blotting to assess overexpression efficiency in keloid fibroblasts. (C) The keloid fibroblasts viability was evaluated by MTT assay. (D–F) The mRNA and protein levels of α‐SMA and COL1A1 in keloid fibroblasts were assessed by qPCR and Western blot analysis. (G) Flow cytometry assay was used to measure the apoptosis rate of keloid fibroblasts. (H) Statistical plot of apoptosis rate of keloid fibroblasts in various groups. (I) Statistical plot of invasion keloid fibroblasts number in various groups (J) The invasion of keloid fibroblasts was determined by Transwell assay. All data are shown as means ± SEM. *N* = 5. One way analysis of variance (ANOVA) followed by Tukey's HSD test were applied for evaluating the significance among multiple groups **p < 0.01.

To investigate the role of RCN1 in keloid fibroblasts, keloid fibroblasts were transfected with pcDNA‐RCN1, and qPCR and Western blotting were used to detect overexpression efficiency. The results showed that overexpression of RCN1 dramatically increased the mRNA and protein levels of RCN1 in keloid fibroblasts (Figure 2A,B and Figure S1A). MTT assay revealed that overexpression of RCN1 markedly promoted cell growth compared to the control (Figure 2C). Overexpression of RCN1 obviously promoted collagen production, manifested by increased mRNA and protein levels of α‐SMA and COL1A1 in keloid fibroblasts (Figure 2D–F and Figure S1B,C). Moreover, flow cytometry analysis indicated that overexpression of RCN1 significantly inhibited the apoptosis of keloid fibroblasts (Figure 2G,H). Transwell assays demonstrated the overexpression of RCN1 also enhanced the invasion of keloid fibroblasts (Figure 2I,J). Collectively, these results illustrated that RCN1 may play a positive role in keloid formation.

### 3.3 ALKBH5 Upregulated RCN1 Expression by Removing m6A Modification

**FIGURE 3:**
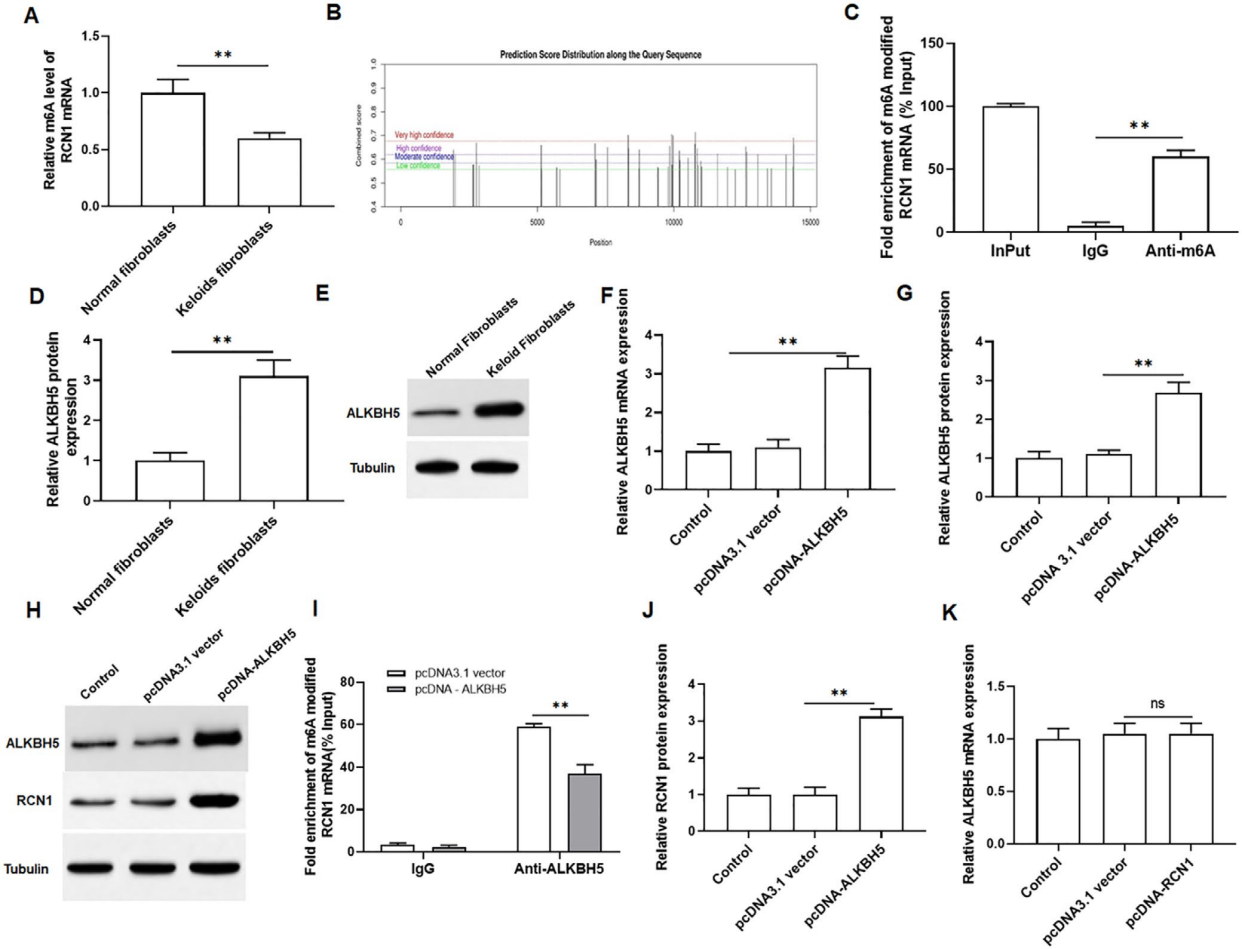
ALKBH5 upregulates RCN1 expression by removing m6A modification. (A) The m6A modified mRNA level of RCN1 in keloid fibroblasts and normal fibroblasts. (B) The specific m6A modification sites of RCN1 were predicted by the SRAMP database. (C) The analysis of m6A level of RCN1 mRNA was detected by MeRIP‐qPCR in keloid fibroblasts. (D, E) The protein expression of ALKBH5 was detected by Western blotting in keloid fibroblasts. Keloid fibroblasts were transfected with pcDNA‐ALKBH5 or pcDNA3.1 vector. (F–H) The mRNA and protein expression of ALKBH5 were detected by qRT‐PCR and Western blotting to assess transfection efficiency. (I) The effect of ALKBH5 overexpression on m6A modification of RCN1 mRNA was detected by MeRIP. (J) Effect of overexpression of ALKBH5 on RCN1 protein expression was evaluated by Western blotting. (K) Effect of overexpression of RCN1 on ALKBH5 protein expression was evaluated by Western blotting. All data are shown as means ± SEM. *N* = 5. One way or Two‐way analysis of variance (ANOVA) followed by Tukey's HSD test was applied for evaluating the significance among multiple groups **p < 0.01.

We assessed the level of m6A modified RNA in normal fibroblasts and keloid fibroblasts using MeRIP, revealing a significant decrease in keloid fibroblasts (Figure 3A). Thus, we predicted the m6A modification site of RCN1 mRNA using the SRAMP prediction server (http://www.cuilab.cn/sramp/), and the results showed that there were many modification sites for RCN1 mRNA (Figure 3B). Then, we performed MeRIP‐qPCR confirmed that overexpression of ALKBH5 reduced the m6A modification level of RCN1 mRNA (Figure 3C). As shown in Figure 3D,E, we observed an increase in the protein expression of ALKBH5 in keloid fibroblasts, indicating that ALKBH5 may play a regulatory role in keloid formation. Additionally, the efficiency of ALKBH5 overexpression was examined, which showed that it significantly promoted the expression of ALKBH5 in keloid fibroblasts (Figure 3F–H). MeRIP‐qPCR was then applied to confirm ALKBH5 mediated m6A demethylation of RCN1 mRNA. Compared to the IgG group (pulldown control), RCN1 mRNA was significantly enriched in RNA‐protein complexes pulled down by m6A specific antibodies. As expected, overexpression of ALKBH5 markedly reduced the m6A level of RCN1 mRNA (Figure 3I). Furthermore, overexpression of ALKBH5 resulted in an increase in RCN1 protein expression (Figure 3J), and overexpression of RCN1 had no effect on the mRNA expression of ALKBH5 (Figure 3K). These findings demonstrated that ALKBH5 upregulates RCN1 expression by removing m6A modifications.

### 3.4 YTHDF2 Recognized the m6A Modification on RCN1 mRNA and Inhibits the Stability of RCN1 mRNA

**FIGURE 4:**
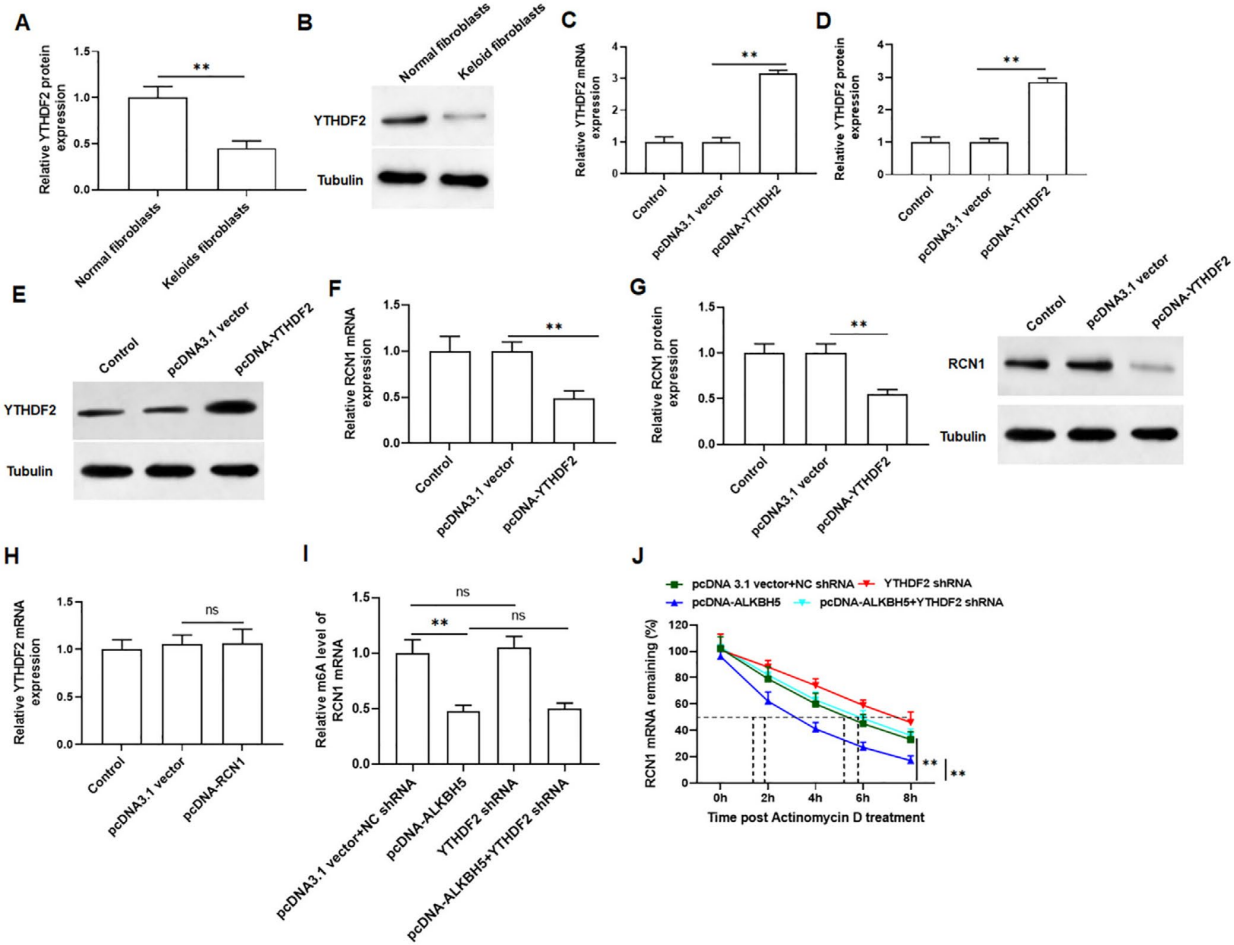
YTHDF2 recognizes the m6A modification on RCN1 mRNA and inhibits the stability of RCN1 mRNA. (A, B) The protein expression of YTHDF2 was detected by Western blotting in keloid fibroblasts and normal fibroblasts. (C–E) The mRNA and protein expression of YTHDF2 were detected by qRT‐PCR and Western blotting to assess transfection efficiency. (F, G) The effect of YTHDF2 overexpression on RCN1 mRNA and protein expression were detected. (H) Effect of overexpression of RCN1 on YTHDF2 protein expression was evaluated by Western blotting. (I) The m6A modified mRNA level of RCN1. (J) Actinomycin D was conducted to evaluate the stability of RCN1 mRNA. All data are shown as means ± SEM. *N* = 5. One way or Two‐way analysis of variance (ANOVA) followed by Tukey's HSD test was applied for evaluating the significance among multiple groups **p < 0.01.

Previous studies suggested that the reader protein YTHDF2 can selectively bind to m6A modified RNAs, recruit them to mRNA decay sites, and then reduce target RNA stability [28, 29]. Therefore, YTHDF2 may be involved in the recognition and regulation of m6A modifications in RCN1 mRNA. We examined YTHDF2 protein expression in normal fibroblasts and keloid fibroblasts, and observed a significant decrease in keloid fibroblasts (Figure 4A,B). Next, we tested the efficiency of YTHDF2 overexpression and showed that overexpression of YTHDF2 markedly promoted the expression of YTHDF2 (Figure 4C–E). As expected, YTHDF2 overexpression markedly reduced the mRNA level of RCN1 in keloid fibroblasts (Figure 4F). Overexpression of YTHDF2 resulted in a decrease in RCN1 protein expression in keloid fibroblasts (Figure 4G), and overexpression of RCN1 had no effect on mRNA expression of YTHDF2 in keloid fibroblasts (Figure 4H). Moreover, we found that overexpression of ALKBH5 reduced the m6A modification level of RCN1 mRNA, while knockdown of YTHDF2 has little effect on the m6A modification level of RCN1 mRNA (Figure 4I). Overexpression of ALKBH5 enhanced the stability of RCN1 mRNA, and knockdown of YTHDF2 inhibited the stability of RCN1 mRNA. Furthermore, knockdown of YTHDF2 reversed the promoting effect of overexpression of ALKBH5 on the stability of RCN1 mRNA (Figure 4J). These results revealed that YTHDF2 is involved in the recognition and regulation of RCN1 mRNA. And it further verified the role of ALKBH5 in regulating the stability of RCN1 mRNA and reveal the combined effect of m6A modification and YTHDF2 knockdown on the stability of RCN1 mRNA.

### 3.5 ALKBH5 Promoted the Proliferation and Invasion, and Inhibited Apoptosis of Keloid Fibroblasts by Regulating m6A Modified RCN1

**FIGURE 5:**
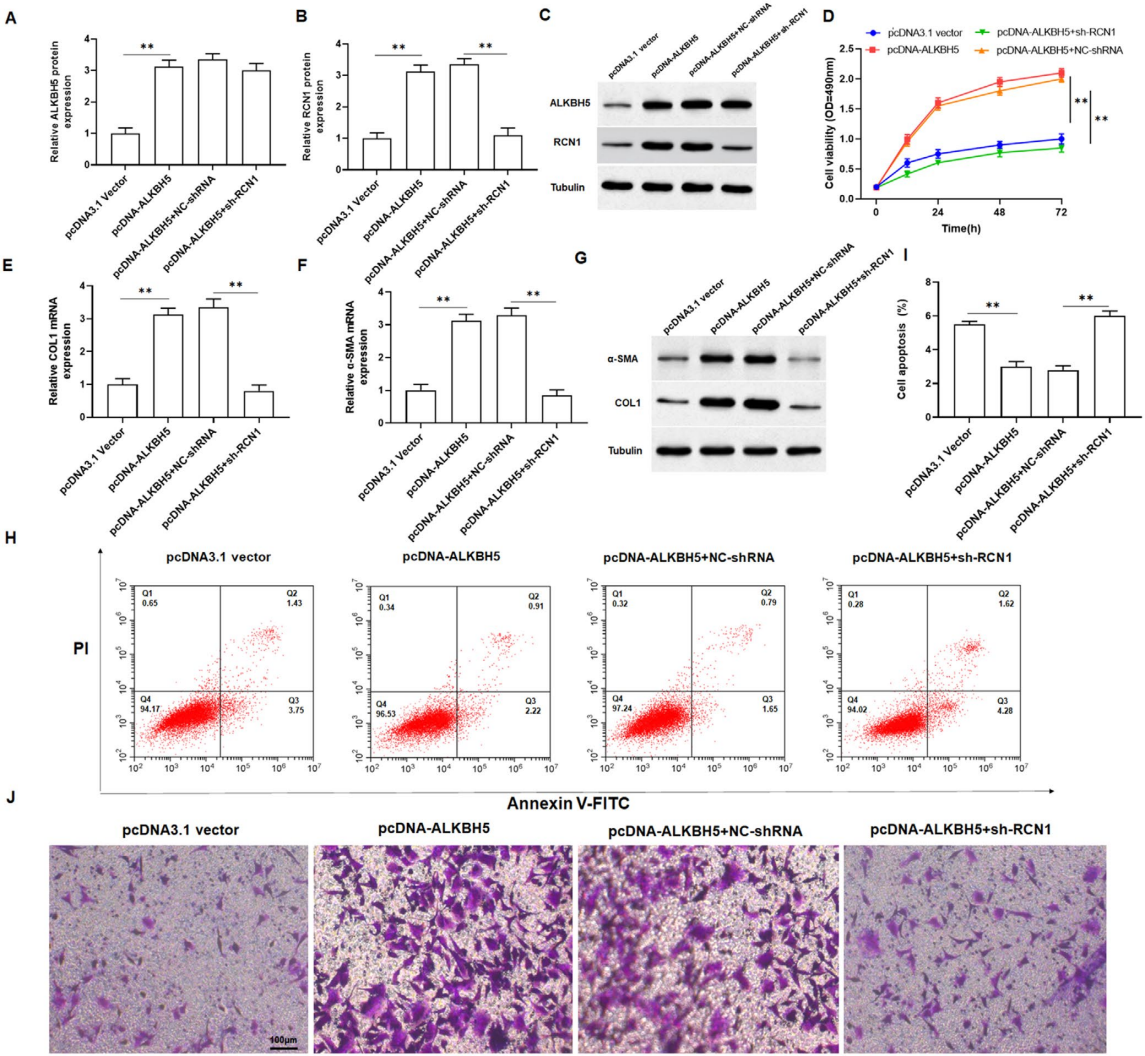
Silencing RCN1 reversed the effect of overexpression of ALKBH5 on keloid fibroblasts. Keloid fibroblasts were transfected with pcDNA‐ALKBH5 or together with RCN1 shRNA or their negative control (pcDNA3.1 vector and NC‐shRNA). (A–C) The protein expression of ALKBH5 and RCN1 were measured by Western blotting to evaluate the transfection efficiency. (D) The viability of keloid fibroblasts was determined by MTT assay. (E–G) qRT‐PCR and Western blot analysis were carried out to assess the α‐SMA, COL1A1 mRNA, and protein levels in keloid fibroblasts. (H) Flow cytometry assay was performed to detect the apoptosis rate of keloid fibroblasts. (I) Statistical plot of apoptosis rate of keloid fibroblasts in various groups. (J) Transwell assay was used to determine the invasion of keloid fibroblasts. All data are shown as means ± SEM. *N* = 5. One‐way analysis of variance (ANOVA) followed by Tukey's HSD test was applied for evaluating the significance among multiple groups **p < 0.01.

To further verify the role of ALKBH5 in keloid formation. we transfected pcDNA‐ALKH5 or co‐transfected with sh‐RCN1 into keloid fibroblasts. Through detecting the protein expression of ALKBH5 and RCN1 in keloid fibroblasts, we confirmed that the transfection of pcDNA‐ALKBH5 and shRNA were successful (Figure 5A–C). MTT assay revealed that overexpression of ALKBH5 significantly promoted the proliferation of keloid fibroblasts, which could be restored by silencing of RCN1 (Figure 5D). qPCR and Western blotting analysis showed that overexpression of ALKBH5 notably promoted the mRNA and protein levels of α‐SMA and COL1A1 in keloid fibroblasts (Figure 5E–G and Figure S2A,B). Furthermore, overexpression of ALKBH5 inhibited cell apoptosis and promoted cell invasion, but this change was abolished with the intervention of knockdown of RCN1 in keloid fibroblasts (Figure 5H–J and Figure S2C). Taken together, these results confirmed that ALKBH5 played a vital role in the regulation of RCN1 in the proliferation, invasion, and apoptosis of keloid fibroblasts.

### 3.6 RCN1 Activated ER Stress to Promote Cell Proliferation and Invasion Through the IRE1α‐XBP1 Signaling Pathway

**FIGURE 6:**
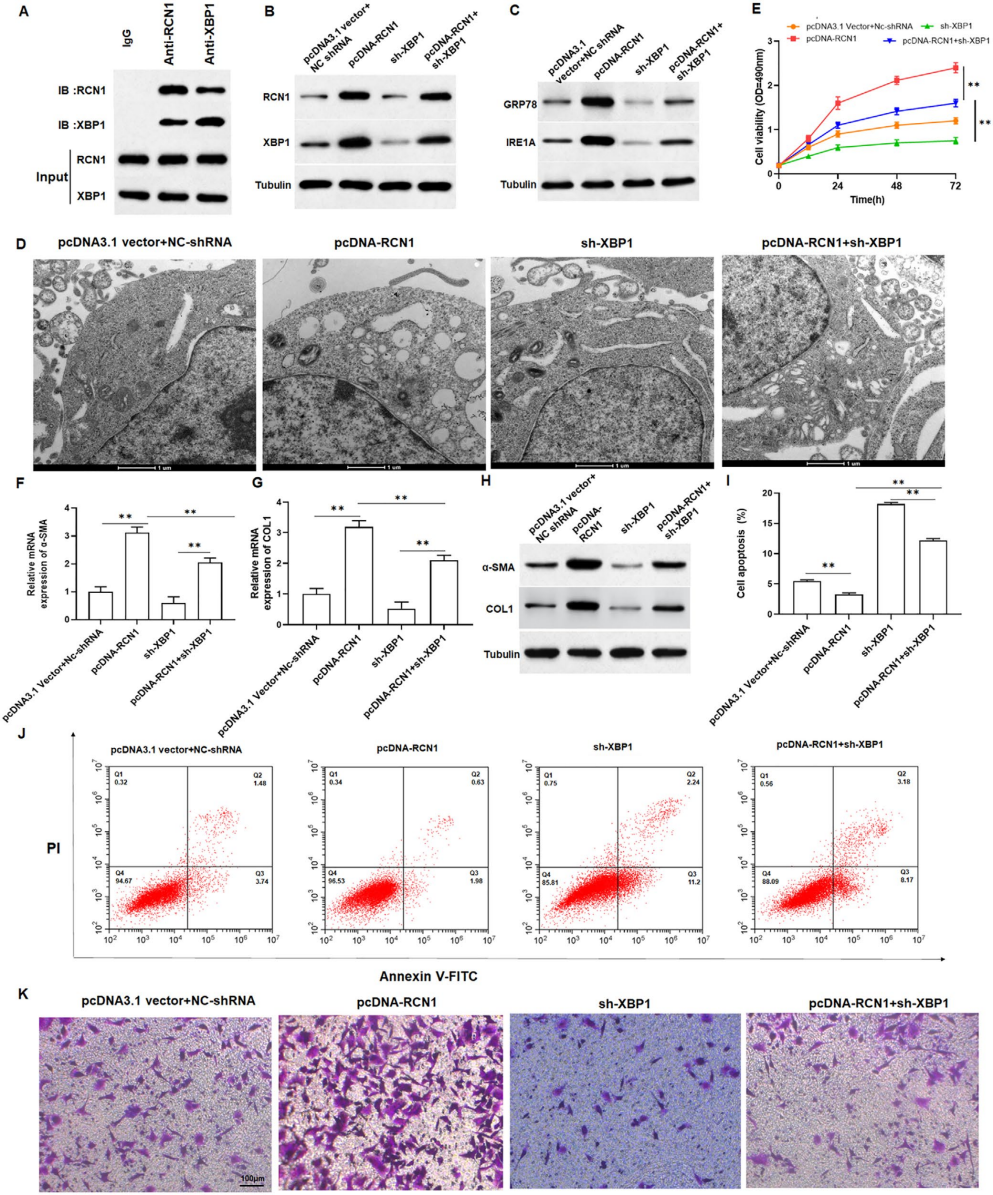
RCN1 activates ER stress to promote cell proliferation and invasion through IRE1α‐XBP1 signaling pathway. (A) Co‐IP was used to verify the interaction between RCN1 and XBP1. Keloid fibroblasts were transfected with pcDNA‐RCN1 and XBP1 shRNA or their negative control (pcDNA3.1 vector and NC‐shRNA). (B) The protein expression of RCN1 and XBP1 were measured by Western blotting to evaluate the transfection efficiency. (C) The protein expression of IRE1α and GRP78 in fibroblasts. (D) The structural changes of ER in fibroblasts were detected by transmission electron microscopy. (E) The viability of keloid fibroblasts was determined by MTT assay. (F–H) qRT‐PCR and Western blot analysis were carried out to assess the α‐SMA, COL1A1 mRNA and protein levels in keloid fibroblasts. (I) Statistical plot of apoptosis rate of keloid fibroblasts in various groups. (J) Flow cytometry assay was performed to detect the apoptosis rate of keloid. (K) Transwell assay was used to determine the invasion of keloid fibroblasts. One‐way All data are shown as means ± SEM. *N* = 5. One‐way or Two‐way analysis of variance (ANOVA) followed by Tukey's HSD test was applied for evaluating the significance among multiple groups **p < 0.01.

Prior report indicated that XBP1 was upregulated in keloid tissues [11]. Firstly, we verified the interaction between RCN1 and XBP1 using Co‐IP. The results showed that XBP1 was present in the samples obtained by immunoprecipitation with an anti‐RCN1 antibody, and RCN1 was present in the samples obtained by immunoprecipitation with an anti‐XBP1 antibody, which illustrated the interaction between RCN1 and XBP1 (Figure 6A). Subsequently, we co‐transfected pcDNA‐RCN1 and sh‐XBP1 into keloid fibroblasts. As shown in Figure 6B and Figure S3A,B, the upregulation of RCN1 significantly promoted XBP1 expression in keloid fibroblasts and sh‐XBP1 reversed the promoting effect of overexpression of RCN1 on XBP1 expression. Thus, we speculated that the role of RCN1 in keloid fibroblasts may be related to ER stress. Meanwhile, TEM results showed that overexpression of RCN1 promoted structural damage and swelling of the ER in keloid fibroblasts (Figure 6D). Compared with the control group, overexpression of RCN1 increased the protein expression of GRP78 and IRE1α (Figure 6C and Figure S3C), promoted cell growth (Figure 6E), collagen production (Figure 6F–H and Figure S3D,E) and invasion (Figure 6K and Figure S3F), and suppressed cell apoptosis in keloid fibroblasts (Figure 6I,J). However, these effects were counteracted by sh‐XBP1. Collectively, overexpression of RCN1 induced ER stress through the IRE1α‐XBP1 signaling pathway.

### 3.7 Silencing RCN1 Inhibited Keloid Formation in Mice

**FIGURE 7:**
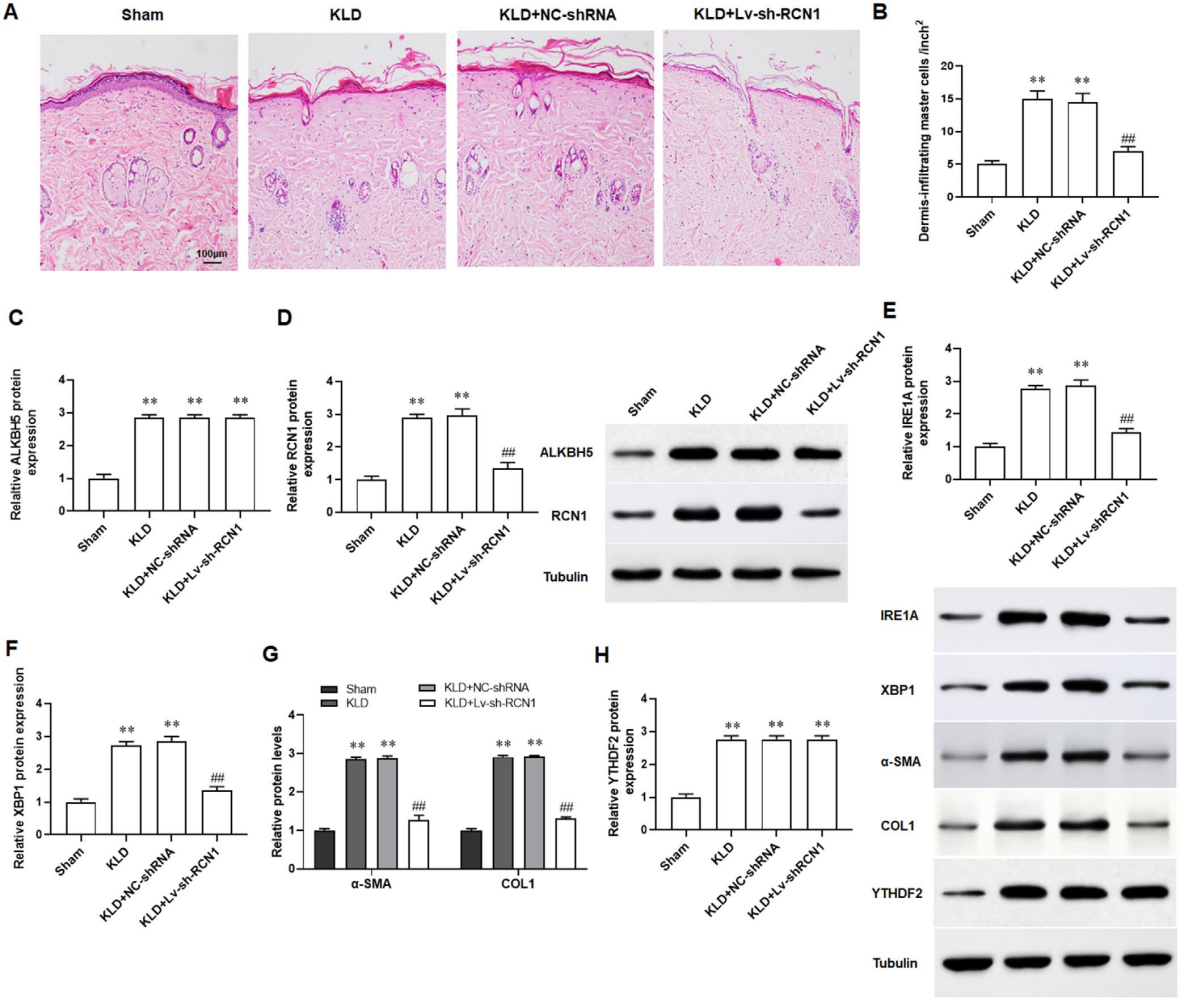
Silencing RCN1 suppresses keloid fibroblasts growth in vivo. These mice were divided into four groups: The sham group, KLD group, KLD + shRNA group, and the KLD+ shRCN1 group. Fibroblasts transfected with LV‐sh‐RCN1 were injected into the skin of mice to establish the model. (A) H&E staining was performed to detect the pathological alterations in keloid skin tissues and normal skin tissues. (B) Statistical dermal mast cell infiltration was used to assess HE‐stained dermal cell hypertrophy and structural abnormalities. (C–F) Western blot analysis was used to detect the protein expression of ALKBH5, RCN1, IRE1α, and XBP1. (G) Western blot analysis was performed to detect the protein expression of α‐SMA and COL1A1. (H) Western blot analysis was performed to detect the protein expression of YTHDF2. All data are shown as means ± SEM. *N* = 12. One‐way or Two‐way analysis of variance (ANOVA) followed by Tukey's HSD test was applied for evaluating the significance among multiple groups. **p < 0.01 compared with the sham group; ##*p* < 0.01 compared with KLD+NC‐shRNA group.

We established a keloid implantation model in BALB/c nude mice using fresh keloid tissues homogenate to assess the effect of sh‐RCN1 on keloid progression in vivo. The results of H&E staining revealed that compared with the skin tissues of normal mice, the dermis of keloid mice exhibited significant hypertrophy and a disordered cellular structure (Figure 7A,B). The results of Western blotting indicated that the protein expression of ALKBH5, RCN1, IRE1α, and XBP1 were increased in skin tissues of keloid mice, and knockdown of RCN1 reversed the changes (Figure 7C–F). And the protein levels of α‐SMA and COL1A1 were found to be upregulated in the skin tissues of keloid mice, and which were restored with the intervention of sh‐RCN1 (Figure 7G). Finally, we found that the expression of YTHDF2 and had no change after silencing RCN1 (Figure 7H). Herein, the keloid implantation model in the nude mice showed that knockdown of RCN1 could suppressed keloid formation in vivo.

## 4 Discussion

In the current study, we probed a novel regulator RCN1 for keloid. RCN1 was upregulated in keloid tissues and keloid fibroblasts compared to normal human skin. Overexpression of RCN1 activates ER stress by regulating IRE1α‐XBP1 pathway and promoting keloid formation. These findings suggested that RCN1 may be a potential therapeutic target for keloid.

RCN1 was upregulated in various cancers, such as glioblastoma, non‐small cell lung cancer and nasopharyngeal carcinoma, indicating its involvement in tumorigenesis and invasion [30, 31, 32, 33]. Knockdown of RCN1 exerts a protective effect against oral squamous cell carcinoma [34]. Downregulation of RCN1 inhibited cell proliferation and promote cell death by activating the AKT and PTEN pathways in prostate cancer cells [13]. Our results suggested that RCN1 was upregulated, and knockdown of RCN1 alleviated keloid formation by inhibiting keloid fibroblasts proliferation, invasion, collagen production and promoting apoptosis.

ER is a multifunctional cellular organelle responsible for the proper folding of newly synthesized proteins, degradation of misfolded proteins, and maintenance of cellular homeostasis [35]. Many genetic and environmental insults, such as disruption of calcium homeostasis, inhibition of protein glycosylation or disulfide bond formation, hypoxia, and the accumulation of unfolded or misfolded proteins in ER lumen, can disturb the function of ER and induce ER stress [36]. Recently, it has been shown that ER stress is involved in the progression of keloid [37]. Prior study noted that TUDCA decreases keloid formation by reducing ER stress as implicated in the pathogenesis of keloid [38]. In addition, IRE1α‐XBP1 was one of the pathways of ER stress and played a key role in keloid development [39]. One study showed that XBP1 along with the UPR signaling pathway were activated in keloid fibroblasts when exposed to a hypoxic environment [40]. Another study suggested that the inhibition of IRE1α also decreased keloid formation and decreased XBP1 [41]. And RCN1 was known to be able to suppress ER stress‐induced apoptosis and is related with tumorigenesis [42]. Our study founded that RCN1 activated ER stress to promote cell proliferation and invasion by activating the IRE1α‐XBP1 signaling pathway.

M6A was considered to be the most abundant internal chemical modification in human mRNA [43]. Owing to recently developed high‐throughput sequencing technology and m6A‐specific antibodies, researchers can precisely determine exact m6A sites and further unravel its functions in both biological, and pathological processes [44]. Recent accumulating evidences indicated that m6A modifications were implicated in keloid [18, 21]. One study suggested that fat mass and obesity‐associated protein (FTO) upregulate COL1A1 expression via regulating COL1A1 m6A modification and maintaining mRNA stability, hence promoting keloid development [23]. Another study confirmed that keloid fibroblasts are in a state of m6A methylation activation, and the high expression of Wnt/β‐catenin pathway in skin fibroblasts, modified and activated via m6A methylation may promote the occurrence of keloid [18]. In addition, ALKBH5 was an important participant in m6A methylation modification [45]. Previous study showed that ALKBH5 inactivated signal transducer and activator of transcription 3 (STAT3) pathway by increasing the suppressor of cytokine signaling 3 (SOCS3) expression via an m6A‐YTHDF2‐dependent manner [46]. And ALKBH5 prevented pancreatic cancer progression by posttranscriptional activation of period circadian regulator 1 (PER1) in an m6A‐YTHDF2‐dependent manner [47]. Moreover, there are reported that ALKBH5 promote the keloid fibroblasts proliferation, migration and invasion [48, 49]. Consistent with these studies, our study suggested that ALKBH5 increased RCN1 expression via an m6A‐YTHDF2‐dependent manner and promoted keloid formation.

However, our study has a couple of limitations. Keloid is a pathological tissue commonly seen in deeply injured humans, but most mammals hardly develop keloid, which makes it challenging to obtain available keloid animal models. Although this experiment utilizes nude mice as an animal model for skin scars, the results obtained from the mouse model may not be entirely applicable to human keloids due to differences in physiological characteristics, metabolic rates, and immune microenvironments between mice and humans. Additionally, the pathological situations in model mice cannot fully stimulate the complexity of human skin wound healing and scar formation. However, the results of this study provide a valuable guidance and reference for clinical practice. Based on this study, targeted treatment plans can be developed to intervene in patients at high risk of pathological scar tissue formation, thereby reducing the likelihood of such formations. In addition, because gene editing technology needs to be carried out in vivo, the location of the nucleus or chromosome, individual genetic differences, potential immune response and other factors may lead to the off‐target of sh‐RCN1. In addition, translating the findings of ALKBH5 and RCN1 into clinical practice may present several challenges. On one hand, how to achieve specific knockdown of ALKBH5 and RCN1 in skin scar fibroblasts from clinical patients is the primary challenge to translate the findings into clinical practice. On the other hand, specific inhibitors of ALKBH5 and RCN1 can be used to improve the progression of skin keloids in clinical patients, but the search for safe and effective targeted specific inhibitors of ALKBH5 and RCN1 is a new challenge. Based on these findings, ALKBH5 and RCN1 can be identified as potential therapeutic targets for skin keloids, and safe and effective targeted specific inhibitors of ALKBH5 and RCN1 can be screened and developed as targeted therapeutic agents for skin keloids to improve keloid formation in clinical patients.

## 5 Conclusion

In this study, we demonstrated that ALKBH5 inhibits YTHDF2‐m6A‐mediated degradation of RCN1 mRNA to promote keloid formation by activating IRE1α‐XBP1‐mediated ER stress. These findings suggested that knockdown of RCN1 may help prevent keloid fibroblasts proliferation and invasion, as well as inhibit keloid progression. Overall, the role of RCN1 described here may enable a deeper understanding of the keloid pathogenesis, thereby benefiting the clinical translation of keloids.

## Author Contributions

Min Shi: conceptualization, investigation, methodology, writing – original draft. Zhuo Zhou: conceptualization, supervision, writing – review and editing. Lu Zhang and Fangfang Bi: methodology, software, visualization.

## Ethics Statement

The study was approved by the Ethics Committee of the Northwest University First Hospital (NUFHLAC‐2022‐056).

## Conflicts of Interest

The authors declare no conflicts of interest.

## Supporting information


Data S1.

## Data Availability

The data that support the findings of this study are available from the corresponding author upon reasonable request.
