# Supplementary material for: ALKBH5 Inhibits YTHDF2‐m6A‐Mediated Degradation of RCN1 mRNA to Promote Keloid Formation by Activating IRE1α‐XBP1‐Mediated ER Stress
Source: J Cosmet Dermatol. 2025 Apr 11;24(4):e70177. doi: 10.1111/jocd.70177 (PMC11987481; doi:10.1111/jocd.70177)
Supplement: Supplementary file 1 — Data S1. [file JOCD-24-e70177-s001.docx]

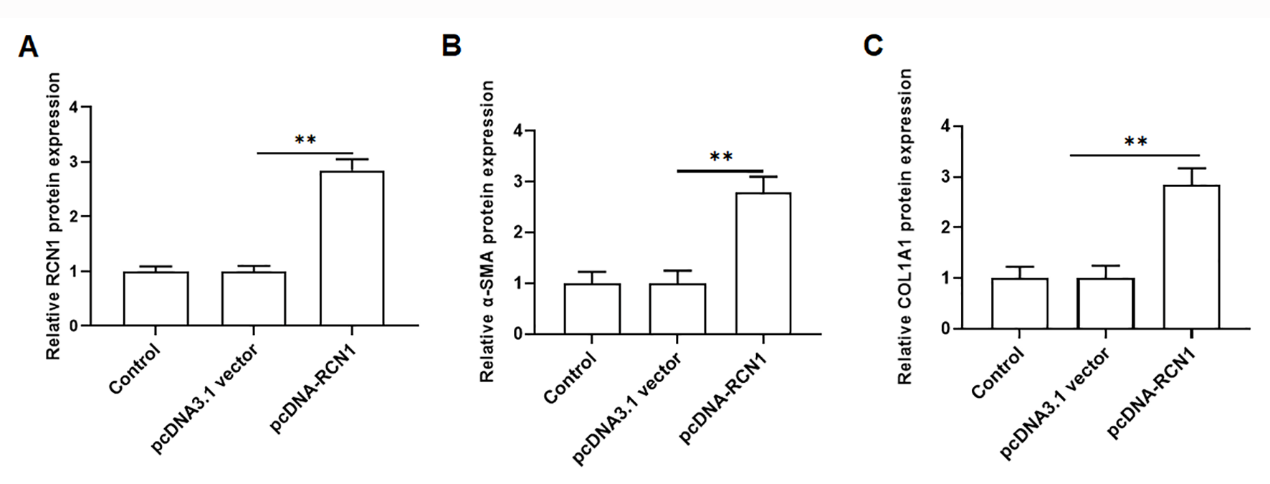


**Supplementary Figure 1** Keloid fibroblasts were transfected with pcDNA-RCN1 or pcDNA3.1 vector. (A) The protein expression of RCN1 was detected by Western blotting in keloid fibroblasts. (B-C) The protein levels of α-SMA and COL1A1 were detected by Western blotting in keloid fibroblasts. One-way or Two-way analysis of variance (ANOVA) followed by Tukey HSD test was applied for evaluating the significance among multiple groups ** *P* < 0.01.

**
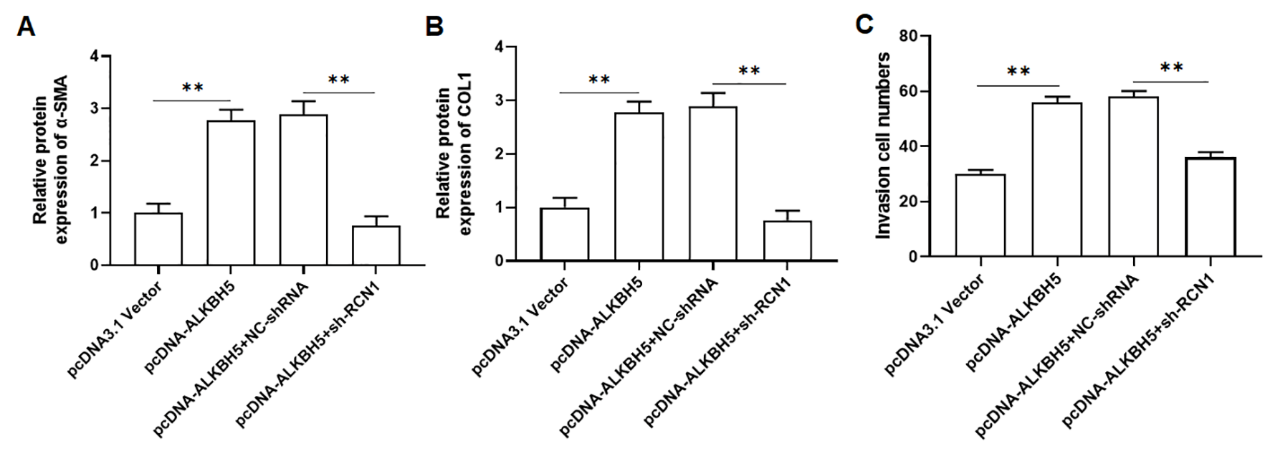
**

**Supplementary Figure 2** Keloid fibroblasts were transfected with pcDNA-ALKBH5 or sh-RCN1. (A-B) The protein levels of α-SMA and COL1A1 were detected by Western blotting in keloid fibroblasts. (C) Statistical plot of invasion keloid fibroblasts number in various groups. One-way or Two-way analysis of variance (ANOVA) followed by Tukey HSD test was applied for evaluating the significance among multiple groups ** *P* < 0.01.

**
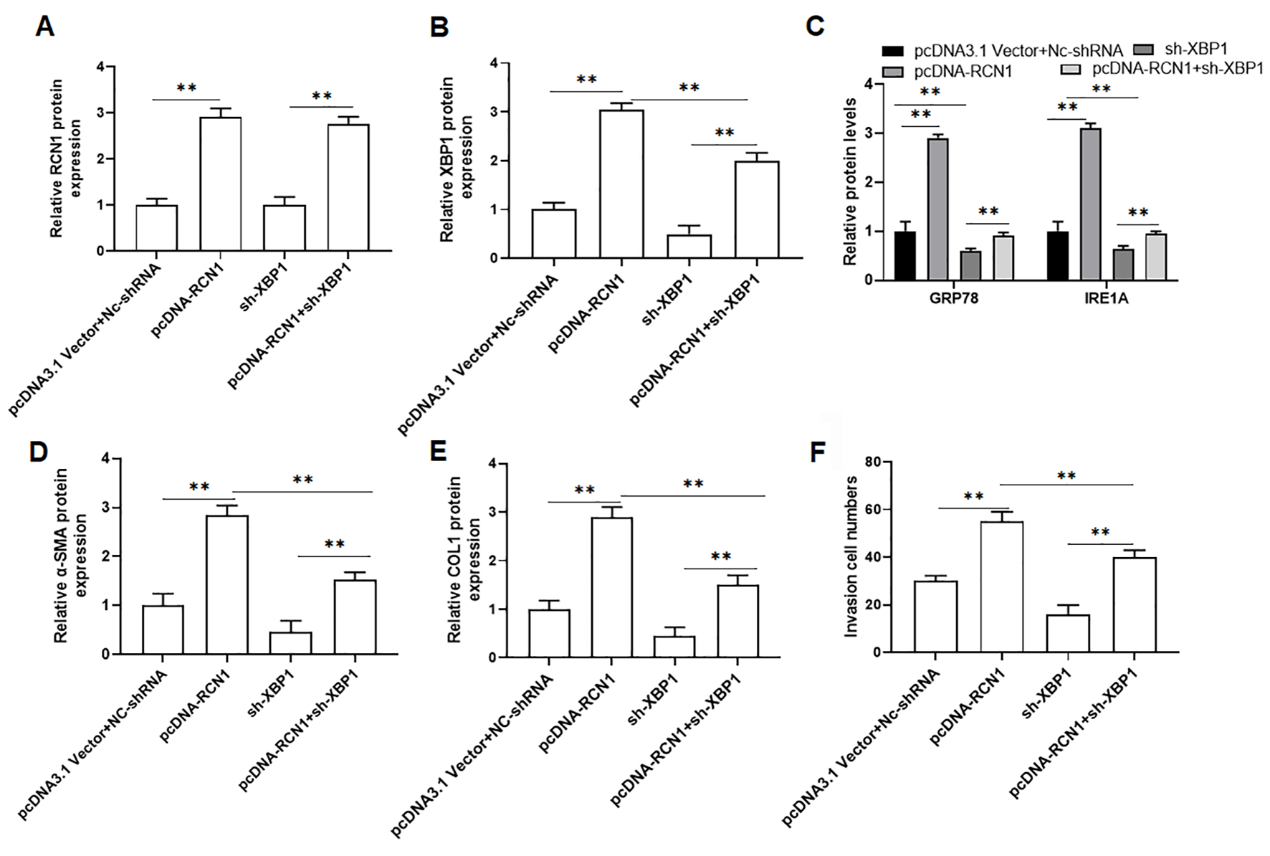
**

**Supplementary Figure 3** Keloid fibroblasts were transfected with pcDNA-RCN1 and XBP1 shRNA or their negative control (pcDNA3.1 vector and NC-shRNA). (A-B) The protein levels of RCN1 and XBP1 were detected by Western blotting in keloid fibroblasts. (C) The protein levels of GRP78 and IRE1α were detected by Western blotting in keloid fibroblasts. (D-E) The protein levels of α-SMA and COL1A1 were detected by Western blotting in keloid fibroblasts. (F) Statistical plot of invasion keloid fibroblasts number in various groups. One-way or Two-way analysis of variance (ANOVA) followed by Tukey HSD test was applied for evaluating the significance among multiple groups ** *P* < 0.01.
